# Crosstalk between proximal tubular epithelial cells and other interstitial cells in tubulointerstitial fibrosis after renal injury

**DOI:** 10.3389/fendo.2023.1256375

**Published:** 2024-01-08

**Authors:** Congcong Guo, Yuying Cui, Mingwen Jiao, Jinming Yao, Junyu Zhao, Yutian Tian, Jianjun Dong, Lin Liao

**Affiliations:** ^1^ Department of Endocrinology and Metabology, The First Affiliated Hospital of Shandong First Medical University & Shandong Provincial Qianfoshan Hospital, Jinan, Shandong, China; ^2^ Shandong Key Laboratory of Rheumatic Disease and Translational Medicine, the First Affiliated Hospital of Shandong First Medical University & Shandong Provincial Qianfoshan Hospital, Jinan, Shandong, China; ^3^ Shandong Institute of Nephrology, the First Affiliated Hospital of Shandong First Medical University & Shandong Provincial Qianfoshan Hospital, Jinan, Shandong, China; ^4^ College of Pharmacy, Shandong University of Traditional Chinese Medicine, Jinan, Shandong, China; ^5^ First Clinical Medical College, Shandong University of Traditional Chinese Medicin, Jinan, Shandong, China; ^6^ Department of General Surgery, The First Affiliated Hospital of Shandong First Medical University & Shandong Provincial Qianfoshan Hospital, Jinan, Shandong, China; ^7^ Department of Endocrinology, Qilu Hospital, Cheeloo College of Medicine, Shandong University, Jinan, Shandong, China

**Keywords:** tubulointerstitial fibrosis, crosstalk, chronic kidney disease, proximal tubular epithelial cells, molecular mechanism

## Abstract

The energy needs of tubular epithelial components, especially proximal tubular epithelial cells (PTECs), are high and they heavily depend on aerobic metabolism. As a result, they are particularly vulnerable to various injuries caused by factors such as ischemia, proteinuria, toxins, and elevated glucose levels. Initial metabolic and phenotypic changes in PTECs after injury are likely an attempt at survival and repair. Nevertheless, in cases of recurrent or prolonged injury, PTECs have the potential to undergo a transition to a secretory state, leading to the generation and discharge of diverse bioactive substances, including transforming growth factor-β, Wnt ligands, hepatocyte growth factor, interleukin (IL)-1β, lactic acid, exosomes, and extracellular vesicles. By promoting fibroblast activation, macrophage recruitment, and endothelial cell loss, these bioactive compounds stimulate communication between epithelial cells and other interstitial cells, ultimately worsening renal damage. This review provides a summary of the latest findings on bioactive compounds that facilitate the communication between these cellular categories, ultimately leading to the advancement of tubulointerstitial fibrosis (TIF).

## 1 Introduction

Approximately 8% to 15% of the global population is impacted by chronic kidney disease (CKD), which has the potential to progress to end-stage renal failure. In such cases, the sole choices for survival are either dialysis or renal transplantation (1). CKD is not just a condition affecting the kidneys, but it is also linked to a higher chance of mortality from any cause and cardiovascular issues (2). Tubulointerstitial fibrosis (TIF), glomerulosclerosis, and vascular sclerosis are common features of progressive CKD, regardless of its initial cause, leading to the development of renal fibrosis. Studies have consistently demonstrated that the increase in TIF is the most reliable histological indicator of renal functional decline and a poorer prognosis (3–6). Despite decades of research, knowledge of TIF remains limited, and therapeutic advances are a daunting challenge (7). Fibrosis progression is contributed to by various cell types found in the tubulointerstitium, such as tubular epithelial cells, fibroblasts, endothelial cells, and inflammatory cells. According to a prior investigation, regions with active interstitial fibrosis were primarily distinguished by their peritubular distribution rather than perivascular distribution (5). The results suggested that tubular epithelial cells can transmit fibrogenic signals, thereby facilitating the advancement of progressive TIF. Additional investigation revealed that proximal tubular epithelial cells (PTECs) were capable of reproducing the histologic alterations in CKD and frequently occurred before the participation of other cellular components. Whether recovery or fibrosis occurs depends on the extent and frequency of PTECs damage. Restoration of normal kidney function is possible if the injury is mild and temporary. Nevertheless, in the case of a severe or persistent injury, the damaged epithelial cells may experience maladaptive healing and produce multiple active substances to trigger secondary responses through paracrine signaling (8). The paracrine signals promote the activation of fibroblasts, the recruitment of inflammatory cells, and the loss of endothelial cells. Hence, the interaction between these different cell types is crucial for the exchange of signals in the tubulointerstitium (9, 10). In the progression of TIF, this review will provide the advanced knowledge regarding the interaction between epithelial cells and other interstitial cells, known as crosstalk.

## 2 Metabolic changes in PTECs associated with survival after injury

**Figure 1:**
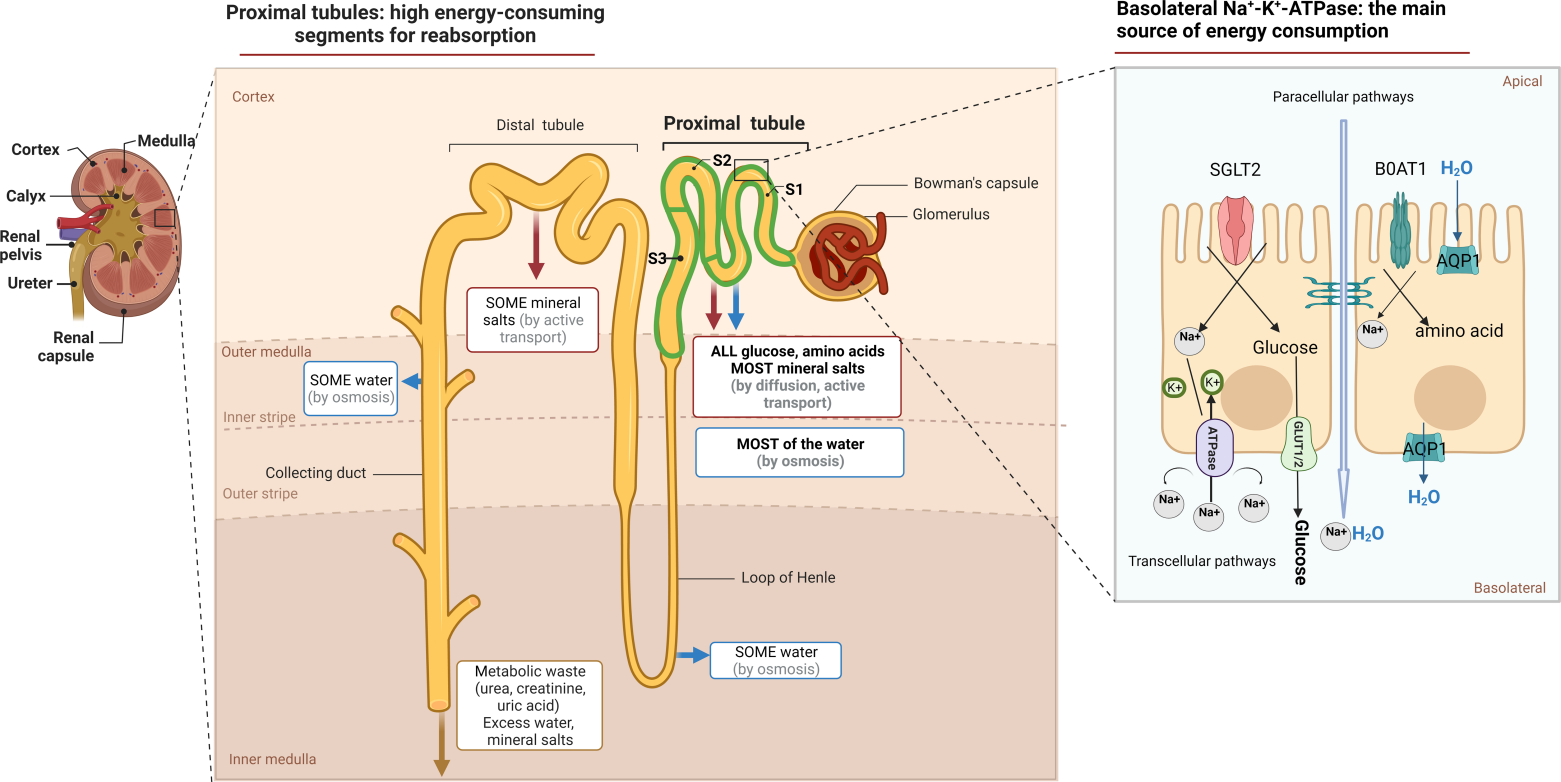
The function and metabolic characteristic of proximal tubule. Anatomically, the proximal tubule refers to the nephron that begins at the urinary pole of the glomerulus and ends at the Henle ‘s loop. Measuring approximately14 mm in length in humans, it constitutes three subtly distinct segments, S1, S2, S3, respectively. The tubular lumen of PTECs is accompanied by a brush border composed of cilia, which not only act as a mechanical sensor during reabsorption, but also increase the cell surface area, maintain normal nephron structure and play a reabsorption function. The reabsorption in the PTs occurs via both paracellular or transcellular pathways. SGLT2 located on the apical surface of the PTECs couples the transport of sodium and glucose in a 1:1 ratio and reabsorbs up to 90% of filtered glucose. The GLUT family located on the basolateral membrane provides an exit pathway for glucose back into the circulation. Various amino acids, similar to that of glucose, are actively transported across the luminal brush border membrane into PTECs, most of which by cotransport. While sodium–glucose or sodium–amino acids linked transport across the apical membrane of the PTECs is not of itself an energy-requiring process, their continuing activity is dependent on the maintenance of the electrochemical gradient for Na^+^, generated by Na^+^/K^+^ ATPase activity.

Physiologically, the proximal tubule (PT), is in charge of preserving 99% of the water and solutes in the glomerular filtrate, while also ensuring the maintenance of acid/base equilibrium (11). Transcellular Na^+^ reabsorption through basolateral Na^+^-K^+^-ATPase is the primary driving force for reabsorption function, as depicted in **Figure 1** (12). The sodium-dependent glucose transporters (SGLT) on the apical surface of PTECs recovers approximately 180 grams of glucose that has been filtered daily. However, little glucose is metabolized as an energetic substrate in PTECs (13). To ensure the energy supply of reabsorption, PTECs use fatty acid oxidation (FAO) as their preferred metabolic pathway, which generates 106 adenosine triphosphate (ATP) units compared to 36 APT units produced by glucose metabolism (14).

**Figure 2:**
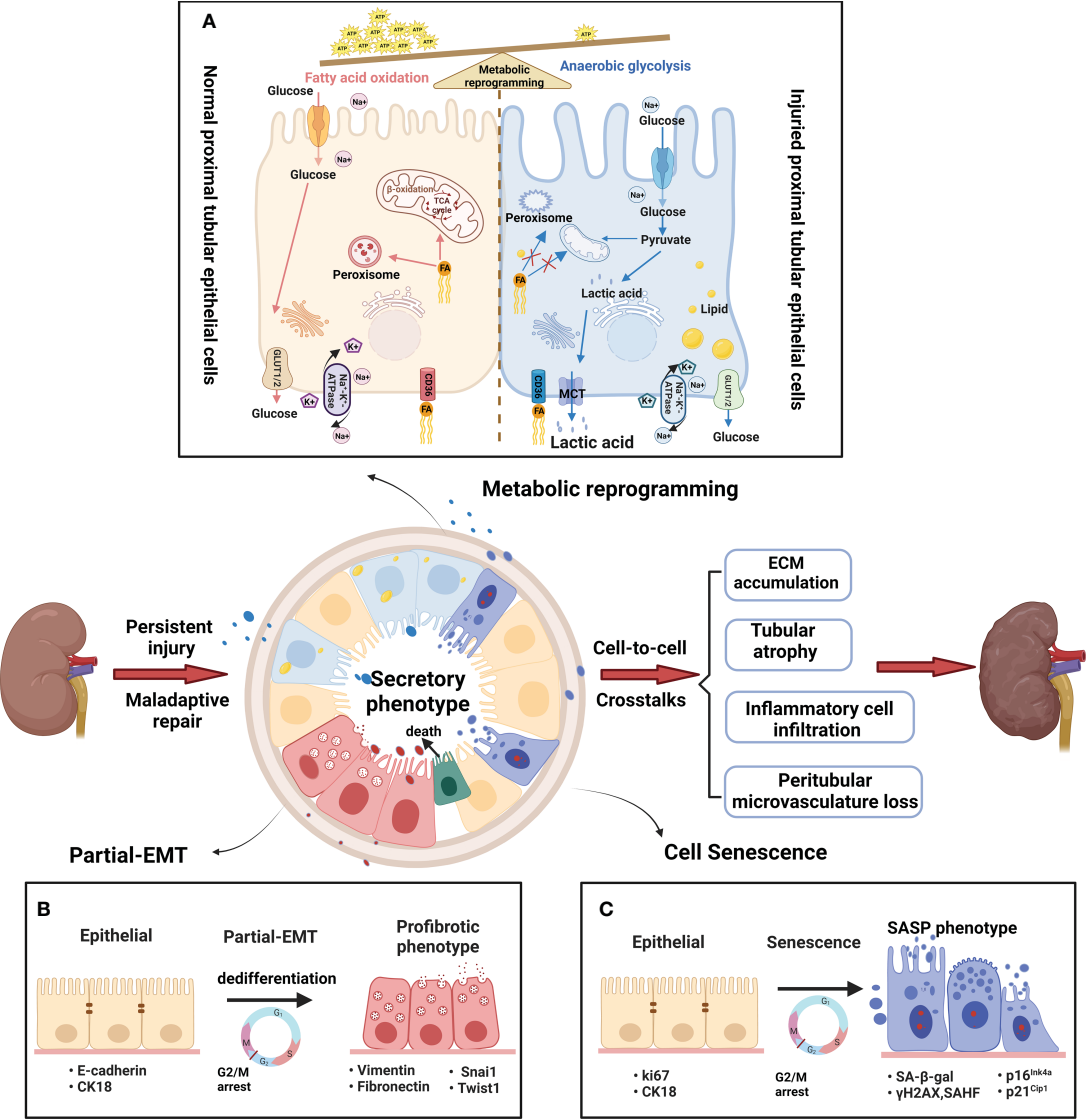
Metabolic and phenotypic changes in proximal tubular epithelial cells. **(A)** Metabolic rearrangement. The normal PTs rely mostly on fatty acid oxidation (FAO) to help meet energetic demands. The fatty acids are absorbed by PTECs through CD36 transporters, and then transported into mitochondria or peroxisomes to generate massive amounts of ATP. A large amount of glucose enters the PTs, but little glucose is metabolized in the normal proximal tubules. By contrast, the injured PTs rely mostly on anaerobic glycolysis to help meet energetic demands. Glycolysis is the process of converting reabsorbed glucose into pyruvate and generating small amounts of ATP. FAO decline due to mitochondrial dysfunction and peroxisome disruption in injured PTs. Therefore, anaerobic glycolysis is used here to the indicate metabolism of glucose to pyruvate and then lactic acid as contrasted with glucose oxidation (pyruvate which then enters the TCA cycle) **(B)** Partial epithelial–mesenchymal transition(pEMT). The pEMT is considered a “dedifferentiation- redifferentiation” process by which PTEC is characterized by the loss of epithelial markers (such as E-cadherin and cytokeratin) and gain of mesenchymal phenotype (vimentin) that is associated with increased production of profibrotic factors (fibronectin). During the process, the pEMT status of PTECs do not convert into interstitial fibroblasts but remain inside the tubule. Snai1 or Twist1 is a key factor in the process. **(C)** Cellular senescence. Cellular senescence is defined as a stable cell cycle arrest coupled with altered transcriptome and secretome. The senescent cells show typical changes with enlarged and flattened cell bodies, increased activity of senescence-associated β-galactosidase (SA-β-gal), and upregulation of cyclin-dependent kinase (CDK) inhibitors, such as p21Cip1 and p16Ink4a. Furthermore, senescent cells acquire senescence-associated secretory phenotype (SASP) to produce a host of proinflammatory and profibrotic cytokines. After persist and repeated injury, the metabolism and phenotype shift result in abnormal amplification of profibrogenic factors via cell-cell crosstalk. Finally, the peritubular microenvironment led to maladaptive repair with tubular atrophy, ECM accumulation, inflammatory cell infiltration and vascular rarefaction.

PTECs, known for their high energy consumption, possess abundant mitochondria, peroxisomes, and other metabolic organelles (15). According to reports, peroxisomes are harmed prior to the onset of mitochondrial dysfunction, making them a probable cause of the observed oxidative stress (16). Acute and chronic injury typically begins at the initial sites of the S2 and S3 proximal segments, likely due to their abundance of peroxisomes (17–19). Furthermore, mitochondrial dysfunction in injured PTECs has been widely reported in both acute kidney injury (AKI) and CKD (20). The process of anaerobic glycolysis can produce 2 ATP molecules without requiring functional mitochondria, resulting in the production of lactic acid. For survival, injured PTECs undergo metabolic reprogramming, which forces a shift from FAO to aerobic glycolysis (**Figure 2A**) (21, 22). In addition, some injured PTECs may undergo apoptosis and necrosis in AKI and CKD (23, 24). Thus, residual PTECs support compensatory changes in reabsorption, along with increased energy expenditure in the short term (25). Abnormal energy supply followed by metabolic rearrangement and increased energy consumption further exacerbate the damage to residual PTECs. FAO downregulation has been described in many AKI and CKD models (14, 26). The ischemia/reperfusion injury (IRI) -induced AKI model (27) and CKD model (28) have both confirmed the beneficial impact of increasing FAO in terms of protection. In recent times, the renoprotective properties of sodium-dependent glucose transporters-2 (SGLT-2) inhibitors on CKD have been shown (29), indicating that the kidney benefits of the drug may be related to decrease energy needs of PTECs and partially depend on reducing glycolysis (30).

## 3 Phenotypic changes in PTECs associated with survival after injury

The partial epithelial-mesenchymal transition (pEMT) alterations and senescence are the main phenotypic changes experienced by PTECs. The introduction of the term “pEMT” was in response to the absence of proof indicating that tubular epithelial cells actively migrate across the basement membrane towards the renal interstitium (31). Residual PTECs within the tubule, as depicted in **Figure 2B**, experience dedifferentiation and enter epithelial G2/M arrest in order to facilitate the replacement of cells that have been lost due to apoptosis/necrosis. Initially, pEMT alterations are adaptive, allowing time for DNA repair and preventing genetic mutations. However, persistent pEMT alterations can promote TIF progression by modifying the epithelial secretome profile (32). The occurrence of accelerated senescence has been noticed in individuals with advanced clinical lesions and even in proteinuric patients who have a normal glomerular filtration rate (GFR), indicating that cellular senescence is an early occurrence in CKD (33). After kidney injury, the PT is typically the primary location for senescent cells (34). Senescence is an intricate and evolutionarily preserved process that has both advantageous and disadvantageous effects, which vary depending on whether the senescent condition is temporary or enduring (35). According to **Figure 2C**, when the senescent state persists, the harmful impacts of senescent cells are strongly linked to the development of a senescence-associated secretory phenotype (SASP). This phenotype generates numerous proinflammatory and profibrotic cytokines, creating an unfavorable microenvironment that contributes to chronic inflammation, adverse tissue remodeling, and fibrosis (36).

The pEMT and cell senescence, both of which involve dedifferentiation, cell cycle changes, and secretory phenotype alteration, have much in common. In fact, pEMT emphasizes phenotypic changes, and senescence emphasizes cell cycle changes. Recent research has affirmed that the inhibition of pEMT is observed when Snai1 or Twist1 is conditionally deleted in PTECs, as indicated by recent studies (32, 37). Silencing p16INK4a and p21Cip1 reduces PTEC senescence, improves tubular regeneration, and attenuates renal fibrosis in AKI and CDK models (38, 39).

## 4 Crosstalk between PTECs and other interstitial cells

Recent data strongly indicate that inhibiting FAO results in a phenotypic change, whereas reversing FAO reduces the impact of this change (40), suggesting that metabolic alterations may occur before phenotypic shifts in PTECs. Initially, the metabolic and phenotypic changes in PTECs in response to sublethal injury are to promote survival and escape cell death. However, this evolutionary adaptation in PTECs cannot be sustained and results in abnormal amplification of profibrogenic factors (32, 35, 41). The paracrine factors can be categorized into three groups based on the substances and size of the particles: microvesicles, exosomes, and paracrine mediators (42). Next, this review focuses on how increasing active substance crosstalk with interstitial cells promotes progressive TIF.

### 4.1 Crosstalk between PTECs and fibroblasts

It is difficult to find myofibroblasts in normal kidneys, however, a significant number of myofibroblasts positive for alpha smooth muscle actin (α-SMA) appear during TIF. Fibroblasts activation into myofibroblasts is considered the primary cells that are responsible for the production of extracellular matrix (ECM). During TIF, PTECs are initial reactors to injury, and myofibroblasts act as final executors. When fibroblasts cannot withstand persistent pathological damage, their roles change from “rescuer” to “destroyer.” The origins of myofibroblasts remain a subject of ongoing investigation. Although myofibroblasts have different origins, there is a widely held belief that resident fibroblasts serve as the precursors for myofibroblasts (43). However, recent studies have shown that marrow-derived fibroblasts contribute to 30% - 60% of myofibroblasts population in the fibrotic kidney, which contribute significantly to the development of renal fibrosis (44, 45). At present, only a few studies have suggested that the recruitment of marrow-derived fibroblasts into the kidney is dependent on the chemokine CXCL16 in tubular epithelial cells (46). However, the activation of marrow-derived fibroblasts may be modulated by T cells. Th2 cells secrete profibrotic factors IL-13 and IL-4 via activating transcription 6 (STAT6) signaling to stimulate marrow-derived fibroblast activation (47). Here, this review focuses on the direct experimental data of the paracrine pathways that mediate communication between PTECs and resident fibroblasts (**Table 1**).

**Table 1:** Summary of mediators that mediate communication between proximal tubular epithelial cells and fibroblasts during kidney injury.

Responding cells	Target cells	Regulators	Mediators	Experimental models	Disease	Main functions	Upstream/Downstream pathway of regulators	References
1.1 PTECs -Derived Communication with Fibroblasts								
1.1.1 Growth and inflammatory factors								
PTECs	Fibroblasts		CTGF ↑	HG treated *in vitro*; mProx24 cells and TFB cells	CDK	Fibroblast activation	**Upstream:** TGF-β in PTECs	(48)
PTECs	Fibroblasts	Genetic deletion of LPA1 *in vivo*;	CTGF ↓	UUO model; LPA1^–/–^ mice; LPA *treated in vitro*; mouse primary proximal tubular epithelial cells;	CKD	Inhibits fibroblast activation and proliferation ECM generation	**Downstream:** LPA-LPA1 signaling induced ROCK activation promoted MRTF-A and -B nuclear translocation and SRF transcriptional activity in PTECs	(49)
PTECs	Fibroblasts	PTEC specific overexpression of EGFR *in vivo*;	CTGF ↑ TGF-β1↑	hHB-EGF^+/Tg^ mice model; hRPTEC/TERT1 cells	CKD	Induces epithelial dedifferentiation and cell-cycle arrest in PTECs; immune cell infiltration; fibroblast activation and proliferation, ECM generation	**Downstream:** Induces p-ERK and SMAD3 activation	(50)
PTECs	Fibroblasts	PTEC-specific knockout of YAP *in vivo*;	CTGF ↑	UNX+STZ induced diabetic model; *Yap^PTiKO^* mice; HEK-293 cells and hRPTC; Mouse or rat fibroblasts;	CKD	Inhibits fibroblast proliferation and activation; ECM generation	**Upstream:** reciprocal interaction between EGFR-PI3K-Akt and RhoA/ROCK signaling pathways in PTECs	(51)
PTECs	Fibroblasts	Tubular HER2 overexpression plasmids *in vivo*;	CTGF ↑	UUO model or STZ-induced diabetic model; NRK52E cells and NRK49F cells	CKD	fibroblast proliferation and activation; ECM generation	**Downstream:** Enhanced p-STAT3 in PTEC; SMAD2 signaling in fibroblast	(52)
PTECs	Fibroblasts	RSL3 treatment and knockdown of GPX4 *in vitro* to induce ferroptosis	CTGF ↑ TGF-β1 ↑ PDGF ↑	UUO model; HK-2 cell; Human kidney fibroblasts	CKD	Decreased cell viability in PTECs; promote ferroptosis profibrotic factors secretion; fibroblast proliferation and activation ECM generation	**Downstream:** ferroptosis in PTEC	(53)
PTECs	Fibroblasts	Tubular INHBB overexpression plasmids *in vivo*; Tubular INHBB overexpression in NEK52E cells	Activin B ↑	UUO model/IRI model;/STZ-induced diabetic model; NRK52E cells and NRK49F cells	CKD	Induce fibroblast activation and proliferation; ECM generation	**Upstream:** SOX9 in PTECs. **Downstream:** activin B/SMAD2 signaling in fibroblasts	(54)
PTECs	Fibroblasts	Genetic deletion of RIG-I *in vivo*; Silencing of RIG-I in HK-2 cells	IL-1β ↓	UUO model/FA models; RIG-I^−/−^mice; Angiotensin II and TGF-β treated *in vitro*; HK-2 cells and NRK49F cells	AKI/ CKD	Suppresses the expression of pro-inflammatory cytokines in HK-2 cells; Suppresses fibroblast proliferation and activation;	**Downstream:** Inhibited c-Myc/TGF-β/SMAD pathway in fibroblasts; NF-κB/IL-6/IL-1β in PTEC	(55)
1.1.2 Wnt and Hh signaling								
PTECs	Fibroblasts	PTEC -specific knockout of β-catenin *in vivo*;	MMP7 ↓	UUO model; Ksp-β-cat^-/-^mice; Recombinant MMP-7 treated *in vitro*; NRK49F cells;	CKD	Reduces EMT in PTECs; Promotes interstitial fibroblast survival;	**Downstream:** Reduce Bax and FasL in fibroblasts	(56)
PTECs	Fibroblasts	PTEC-specific overexpression of EGFR *in vivo*; Recombinant Wnt1 treated with NRK-49F cells;	Wnt1 ↑	Bigenic SLC34a1^GCE/+^; R26^Wnt1-GFP/+^ mice; TGF-β treated *in vitro*; NRK-49F cells;		Increase TGF-β expression; Promotes fibroblast proliferation and activation; ECM generation;	**Downstream:** Wnt1/β-catenin interact with TGF-β-SMAD3 signaling in fibroblasts	(57)
PTECs	Fibroblasts	Shh overexpression plasmid *in vivo*;	Shh ↑	IRI model/ADR model; Gli1-CreER^T2^ transgenic mice; NRK-49F cells and HKC-8 cells,	AKI	Promotes fibroblast proliferation and activation; ECM generation; Accelerates AKI to CKD Progression	**Downstream:** Gli1 in fibroblasts	(58)
PTECs	Fibroblasts	Recombinant Shh treated with NRK49F cells	Shh ↑	UUO model; Gli1^lacZ^ mutant mice; NRK-49F cells	CKD	Induce fibroblast proliferation and activation; ECM generation	**Downstream:** Gli1 and Snail1 in fibroblasts	(59)
1.1.3 Exosomes and Extracellular vesicles								
PTECs	Fibroblasts		TGF-β1 mRNA in exosomes ↑	UUO model; Hypoxia treated *in vitro*; HK-2 cells, NIH-3T3 cells and TFBs cells,	CKD	Promotes fibroblast proliferation and activation, ECM generation		(60)
PTECs	Fibroblasts		22 proteins in exosomes↑	Akita diabetic model; C57BL/6J-Ins2 Akita/+ mice; HG treated *in vitro*; BUMPT cells and NRK-49F cells	CDK	Promotes fibroblast activation and proliferation, ECM generation		(61)
PTECs	Fibroblasts	Aldosterone treated *in vivo* and *in vitro*;	Evs miR-196b-5p ↑	db/db model; HK-2 cells; CCD-8Lu cells;	CKD	Induce PTECs injury; Promoted fibroblast activation and proliferation; ECM generation;	**Upstream:** Activate MR in PTECs **Downstream:** SOCS2 inhibition and enhanced p-STAT3 in fibroblasts.	(62)
PTECs	Fibroblasts	GW4869 treatment *in vivo* and *in vitro*; Rab27aknockdown *in vitro*;	Exosomal miR-150 ↓	IRI model; Hypoxia treated *in vitro*; NRK-52E cells and NRK-49Fcells	AKI	Inhibits proliferation and promotes apoptosis of fibroblast; ECM generation; Ameliorates renal fibrosis		(63)
PTECs	Fibroblasts	Genetic deletion of Rab27a or GW4869 treatment *in vivo*	Exosomal miR-150-5p ↓	UIRI model; Rab27a knockout mice; Hypoxia treated *in vitro*; NRK-52E cells and NRK-49Fcells	AKI	Inhibition of exosome secretion; attenuates fibroblast activation and proliferation, ECM generation	**Downstream:** SOCS1 inhibition in fibroblast	(64)
PTECs	Fibroblasts	Genetic deletion of Rab27a *in vivo*;GW4869 treatment in NRK-52E cells	Exosomal miR-21 ↓	UUO model; Rab27a knockout mice; TGF-β1-treated *in vitro*; NRK-52E cells and NRK-49Fcells	CKD	Inhibition of exosome secretion; attenuates fibroblast activation and proliferation, ECM generation	**Downstream:** PTEN/Akt pathway in fibroblasts	(65)
PTECs	Fibroblasts	Downregulate miR-503-5p and GW4869 treatment in NRK-52E cells	wnt1, wnt4 and miR-503-5p in exosome ↓	Cadmium-induced kidney fibrosis model; Cadmium treated *in vitro*; NRK-52E cells and NRK49F cells	CKD	Promoted EMT in PTECs; fibroblast activation; ECM generation;	**Downstream:** Enhance β-catenin pathway in PTECs and fibroblasts	(66)
1.1.4 Lactic acid								
PTECs	Fibroblasts		Lactic acid ↑	FA model; Primary tubular cells and NRK-49F cells	AKI	Induce fibroblasts activation and proliferation; ECM generation	**Downstream:** Induce HGF expression in fibroblasts	(67)
1.2 Fibroblasts-Derived Communication with PTECs								
Fibroblasts	PTECs		Mvs miR-34 ↑	UUO model; TGF-β1 treated *in vitro*; NRK-52E cells and NRK-49Fcells	CKD	Induces tubular epithelial cells apoptosis	**Downstream:** Downregulated the expression of Bcl-2 in PTECs	(68)
Fibroblasts	PTECs		Mvs miR-34 ↑	UUO model; TGF-β1-treated *in vitro*; NRK-52E cells and NRK-49F cells	CKD	Induces tubular epithelial cells apoptosis	**Downstream:** Downregulated the expression of Bcl-2 in PTECs	(69)
Fibroblasts	PTECs	ICG-001 treatment *in vivo*; Fibroblast-specific ablation of β-catenin *in vivo*;	HGF ↑	IRI mice; Gli1-β-cat^-/-^ mice; Ksp-β-cat^-/-^ mice; Recombinant Wnt treated *in vitro*; HKC-8 cells and NRK-49Fcells	AKI	Reduces PTECs apoptosis; Restored renal Na^+^/K^+^-ATPase; Promotes Tubular Regeneration; Attenuates Renal Inflammation after AKI	**Downstream:** Promotes HGF/c-met Signaling	(70)
Fibroblasts	PTECs	Fibroblast-specific ablation of Rheb and Rictor to blockade of mTORC1 and mTORC2 signaling *in vivo*	HGF ↓	IRI model; Staurosporine-treated *in vivo*; NRK-49Fcells;NRK-52E cells; Primary fibroblasts from Rheb^fl/fl^,Tsc1^fl/fl,^ and Rictor^fl/fl^ model	AKI	Induce tubular cells survival death; aggravates kidney	**Downstream:** Downregulated mTOR/PPARγ/HGF axis in fibroblast; c-met signaling in PTECs	(71)
Fibroblasts	PTECs	Fibroblasts- specific overexpression of p90RSK *in vivo*;	H_2_O_2_ ↑	UUO model; FSP-1-specific p90RSK transgenic mice; HKC-8 cells and primary fibroblasts isolated from p90RSK mice and wide type	CKD	Induce tubular epithelial cells apoptosis	**Downstream:** Activates ROS/β-catenin or FOXO1 activity in PTECs	(72)
1.3 Reciprocal activation loop between PTECs and activated fibroblasts								
PTECs Fibroblasts	Fibroblasts PTECs	pFlag-Wnt9a plasmid *in vivo*; Recombinant Wnt9a treated in HK-2cells and NRK4F cells	TGF-β1 ↑ Wnt9a ↑	UIRI model/UUO model/ADR nephropathy model; Overexpression of Wnt9aC57BL/6 model; HK-2 cells; primary mouse tubular cells and NRK-49F cells	CKD	Induce fibroblast activation and proliferation; cellular senescence in PTECs; ECM generation	**Downstream:** Activates P16^INK4A^ in PTECs	(73)
PTECs Fibroblasts	Fibroblasts PTECs	Tonabersat blocks Cx43 hemichannel in HK-2 cells and TK173 cells	ATP ↓	TGF-β1/HG-treated; HK-2 cells and TK173 cells	CKD	Inhibits TGFβ1/HG -evoked increases in Cx43, adherens junction and ECM protein expression in HK-2cells and TK173 cells	**Downstream:** Activates P2X7 receptor in fibroblasts and tubular cells	(74)

#### 4.1.1 PTECs-derived communication with fibroblasts

##### 4.1.1.1 Growth and inflammatory factors

The advancement of TIF has been linked to the involvement of transforming growth factor (TGF-β), connective tissue growth factor (CTGF), and platelet-derived growth factor (PDGF) (10). Furthermore, apart from alterations in metabolism and phenotype, ferroptosis-mediated PTEC death triggers the stimulation of neighboring fibroblasts through the enhancement of the paracrine function of pro-fibrotic substances (TGF-β1, CTGF, and PDGF) within PTECs (53). TGF-β1, out of the three TGF-β isoforms (β1, β2, β3), is regarded as a dominant initiator because it can trigger the transformation of fibroblasts into myofibroblasts and promote the production of ECM (75, 76). It is now well accepted that both canonical (SMAD-dependent) and non-canonical (SMAD-independent) signaling pathways serve as the two major downstream pathways that promote TGF-β1-mediated myofibroblast activation (77). Latency-associated peptide (LAP) forms complexes with TGF-βs when they are secreted, rendering them inactive. Integrin αvβ6 binds to TGF-β1 LAP. The hidden TGF-β1 complexes attach to αvβ6, enabling fully developed TGF-β1 to reach TGF-β receptors (TβRII) and initiate the traditional TGF-β signaling (78). Furthermore, empirical proof indicated that the transmission of TGF-β mRNA into fibroblasts through exosomes was an alternative method to trigger TGF-β activity (60). Despite the strong association between renal fibrosis and TGF-β1, attempts to treat diabetic nephropathy using clinical trials focused on TGF-β1 or neutralizing antibodies against it have not yielded any positive outcomes (79). Possible explanations for these results are that other growth factors may have negated its benefit. For example, apart from TGF-β1, activin B derived from tubules (which is a part of the TGF-β family) is involved in the development of renal fibrosis by stimulating neighboring fibroblasts through paracrine signaling in a unilateral ureteral obstruction (UUO) model (54). CTGF (also referred to as CCN2) is another growth factor that has a significant impact on the reaction to long-term kidney damage. Several signaling pathways in damaged PTECs can mediate CTGF expression in CKD. Persistent activation of YAP (yes-associated protein)) (51), HER2 (human epidermal growth factor receptor 2) (52), EGFR (epidermal growth factor receptor) (50) and the LPA-LPA1 signaling pathway (49) in tubular cells is required for activating fibroblasts by inducing CTGF.CTGF serves as a partner for TGF-β.CTGF does not possess a specific receptor, however, it seems to engage with TβRII in fibroblasts to facilitate the development of a pro-fibrotic atmosphere. CTGF has the ability to attach to lipoprotein receptor-related proteins 6 (LRP6), which results in the activation of β-catenin and the promotion of TIF (80). Patients with diabetic nephropathy exhibit increased levels of CTGF in both their plasma and urine (81, 82). TGF-β by not PDGF mediates high glucose induction of CTGF expression in PTECs. Moreover, damaged PTECs within fibrotic tissue exhibited heightened TGF-β functionality, indicated by the phosphorylation of downstream Smad2, alongside elevated CTGF levels (83). The findings from these studies suggest that several growth factors are crucial in facilitating the progression of CKD, and focusing solely on one growth factor is inadequate to modify the trajectory of TIF.

Besides the growth factors, damaged PTECs can trigger the inflammatory response by inducing various inflammatory factors such as interleukin (IL)-1β and IL-6. Multiple studies have shown that retinoic acid-inducible gene-I (RIG-I), implicated in numerous chronic inflammatory conditions (84), plays a crucial role in a proinflammatory pathway (85). In unilateral UUO and folic acid renal fibrosis models, retinoic acid-inducible gene-I (RIG-I) functions as a promoter of NF-κB (nuclear factor-kappa B) signaling, leading to the activation of NF-κB signaling and facilitating the production and secretion of IL-1β and IL-6 in PTECs. The release of proinflammatory cytokines by PTECs greatly increases the expression of c-Myc in fibroblasts, leading to the activation of TGF-β/Smad signaling-mediated fibroblast activation and the synthesis of ECM components (55).

##### 4.1.1.2 Wnt and Hh signaling

Wnt/β-catenin signaling remains inactive in normal adults, however, it gets reactivated in the kidneys following both acute and chronic damage. The Wnt ligands consist of a group of proteins that are secreted to transmit signals. Humans have a total of 19 members belonging to the WNT family, including WNT1, WNT2, WNT2B, WNT3, WNT3A, WNT4, WNT5A, WNT5B, WNT6, WNT7A, WNT7B, WNT8A, WNT8B, WNT9A, WNT9B, WNT10A, WNT10B, WNT11, and WNT16. Each one has the ability to attach to the Frizzled and LRP5/6 (lipoprotein receptor-related proteins 5 and 6) receptors, resulting in the activation of canonical signaling pathway via β-catenin nuclear translocation and the increase in expression of Wnt target genes. Numerous researches have indicated that the timely and suitable initiation of Wnt/β-catenin signaling is commonly believed to have a protective effect (86). For example, in AKI models like IRI and folic acid nephropathy, the elimination of β-catenin specifically in the tubules has demonstrated the worsening of kidney damage through the elevation of PTEC apoptosis (87). In stark opposition to the detrimental impact of tubule-specific elimination of β-catenin, the elimination of β-catenin in fibroblasts protects kidneys from tubular apoptosis and hinders renal inflammation in AKI (70). In the CKD model, the depletion of β-catenin in the tubules does not have a significant impact on the apoptosis of tubular cells, which contradicts its function in AKI (56). There was no variation in the severity or extent of renal fibrosis after UUO when β-catenin depletion occurred, however, it enhanced the survival of interstitial fibroblasts through the MMP-7 (matrix metalloproteinases-7)/FasL (Fas ligand) pathway (56). The difference might be associated with the unique characteristics of the injuries in AKI compared to CKD. Unlike the beneficial transient activity in acute injury, continuous stimulation of Wnt signaling in PTECs is harmful and triggers epithelial pEMT, along with unregulated fibroblast activation and excessive accumulation of ECM in long-term harm (57, 88). Collectively, these findings indicate that the function of Wnt/β-catenin activation is influenced by both the stage of the disease (AKI versus CKD) and the specific tissue components (renal tubules versus fibroblasts).

Similar to Wnt/β-catenin signaling, Hedgehog (Hh) signaling controls tissue arrangement, differentiation, and cellular proliferation. Shh, one of the three Hh ligands (including Dhh and Ihh), is the most extensively studied. Shh binds to the Patched1 receptor on the membrane leads to the release of the protein smoothened (Smo), which in turn promotes the transcription of Gli1/2 effectors. The immunohistochemical staining revealed that the Shh protein was primarily found in the tubular epithelial cells, while the interstitial cells showed mostly no signs of Shh staining. Fascinatingly, only fibroblasts in the UUO model expressed the receptor Patched1 and the Gli transcriptional targets (59). In different animal models of CKD, PTECs are primarily responsible for the induction of Shh, which specifically stimulates fibroblast proliferation and worsens fibrotic kidney lesions (58). Hence, the signaling pathway of Shh/Gli1 plays a vital function in facilitating communication between epithelial cells and fibroblasts in the process of renal fibrogenesis. In the treatment of various fibrotic kidney disorders, Targeting Shh/Gli1 signaling could be considered as a new strategy.

##### 4.1.1.3 Exosomes and extracellular vesicles

Exosomes and extracellular vesicles (EVs) have the ability to effectively transport bioactive substances, such as miRNAs, proteins, and mRNA, to facilitate the exchange of genetic information between cells (89). According to a recent investigation, twenty-two proteins discovered in tubular exosomes have the ability to facilitate tubulointerstitial communication and control the advancement of renal fibrosis in DKD (61). Exosomes can transport hypoxia-induced TGF-β mRNA, which then stimulates fibroblasts, thereby playing a role in the progression of renal fibrosis (60). Small noncoding RNAs called miRNAs control gene expression by binding target mRNAs, leading to degradation or translational repression. In addition to directly controlling the expression of target genes in target cells, miRNAs can also regulate the expression of target genes in different cells by delivering them through exosomes. After an ischemia‒reperfusion incident, exosomes derived from PTECs stimulate fibroblast activation and proliferation through the action of miR-150-5p and miR-150 (63, 64). MiR-21, extensively researched, is known to be involved in the control of fibroblast proliferation (90). *In vitro*, the activation of fibroblasts was expedited by miR-21-containing exosomes derived from epithelial cells, while *in vivo*, the presence of miR-21-deficient exosomes alleviated renal fibrosis after UUO by acting on the PTEN/Akt pathway (65). Rab27a, a member of the RAB GTPase family, plays a significant role in the production and release of exosomes (91). Multiple studies suggest that Rab27a knockout or GW4869 (exosome secretion inhibition) treatment can ameliorate renal fibrosis in AKI and CKD models (64, 65). In addition to profibrotic miRNAs, PTECs-derived exosomal miR-503-5p protects against cadmium nephrotoxicity and is capable of suppressing pEMT and fibroblast activation (66). Novel mediators of cellular crosstalk are emerging in the form of PTEC-derived EVs, which have a larger volume compared to exosomes (92). In the study conducted by Renzhi Hu, it was found that miR-196b-5p (EVs) facilitate communication between proximal tubular epithelial cells (PTECs) and fibroblasts in the development of renal fibrosis caused by aldosterone in individuals with diabetes. This communication is achieved by suppressing the expression of SOCS2 and promoting the phosphorylation of Stat3, as stated in the research (62).

##### 4.1.1.4 Lactic acid

Lactic acid, the final product of glycolysis, has been traditionally considered a metabolic byproduct. Recently, the role of lactic acid in cell proliferation has gained significant attention (93). Fibroblasts take up lactate excreted by tubules via monocarboxylate transporter-1 (MCT1). Tubule-derived lactic acid is required for fibroblast proliferation, which is considered to facilitate repair in the early phase of AKI (67). This protective effect of lactic acid could potentially be linked to instructing fibroblasts in generating hepatocyte growth factor (HGF) (67, 94). However, in a CKD model, inhibiting aerobic glycolysis in PTECs suppressed fibroblasts from transforming into matrix-producing myofibroblasts (95). These results suggest the differential roles of lactic acid in different stages of disease progression.

#### 4.1.2 Fibroblasts-derived communication with PTECs

When faced with injury, certain PTECs undergo apoptotic death, resulting in the destruction of the kidney parenchyma. Initially, fibroblasts create a conducive atmosphere to facilitate the regeneration of PTECs in response to temporary damage. HGF, a protein with renoprotective properties, is synthesized by fibroblasts that binds to the c-met receptor found on both PTECs and fibroblasts. The downstream effects of HGF (hepatocyte growth factor)/c-met (cellular-mesenchymal epithelial transition factor) signal transduction include cell survival, proliferation, differentiation, and migration (96). In an AKI model, activation of mTORC1 (mammalian target of rapamycin complex 1) and mTORC2 (mammalian target of rapamycin complex 2) signaling in fibroblasts safeguards against ischemia/reperfusion-induced PTECs death by inducing HGF. β-Catenin is an upstream negative regulator of HGF. In AKI, the reduction of PTECs apoptosis and the promotion of regeneration are achieved by increasing HGF expression through the specific elimination of β-catenin in fibroblasts (70, 71). After kidney injury, tubules serve as the primary origin of Wnt ligands (97). As mentioned previously, overexpressed Wnt1 in PTECs could specifically target fibroblasts, resulting in the activation of β-catenin (57). Consequently, it suppresses the expression of HGF in fibroblasts and lead to PTECs apoptosis. The findings suggest that the interaction between Wnt and HGF signaling facilitates the bidirectional communication between PTECs and fibroblasts, playing a crucial role in promoting TIF progression.

The UUO model demonstrates that the stimulation of 90 kDa ribosomal protein S6 kinase 1 (p90RSK) triggers kidney fibrosis by causing fibroblast-induced PTECs apoptosis through the activation of the β-catenin/FOXO1 signaling pathway via ROS (98). The secretion of detrimental molecules through microvesicles was previously reported to be a self-defense strategy in cells (99). In the CKD model, miR-34a was reported to promote cell apoptosis by inhibiting the anti-apoptotic Bcl-2, which was upregulated in the fibroblasts. The injured fibroblasts secrete miR-34a via microvesicles to avoid apoptosis. Nonetheless, the miR-34a carried in microvesicles subsequently disrupts the tubular basement membrane and triggers PTECs apoptosis by specifically targeting Bcl-2 (B-cell lymphoma-2) (68, 69). These findings suggest that persistent harmful triggers in CKD can lead to extensive activation of signaling agents and disrupt the defensive impact of fibroblasts, leading to progressive tissue damage and functional decline.

#### 4.1.3 Reciprocal activation between PTECs and fibroblasts

The pro-fibrotic microenvironment is facilitated by a reciprocal activation loop between senescent/pEMT epithelial cells and activated fibroblasts. Recently, experimental data further confirmed that Wnt9a is involved in regulating communication via paracrine or autocrine mechanisms. The induction of senescent tubular cells by Wnt9a resulted in the secretion of TGF-β1, a component of the SASP. This secretion subsequently caused the proliferation of interstitial fibroblasts and the transformation into myofibroblasts. Subsequently, TGF-β1 stimulated the expression of Wnt9a in fibroblasts, resulting in the establishment of a mutual activation cycle between senescent tubular cells and activated fibroblasts (73). Connexin 43 (Cx43) is a connexin (Cx) that constitutes gap junctions. Adjacent cells can directly exchange small signaling molecules. *In vitro*, Cx43 and P2X7R are critical factors contributing to epithelial-fibroblast communication circuits. Blocking Cx43-mediated hemichannel ATP release can decrease P2X7 receptor (P2X7R) activation in PTECs and fibroblasts, thus preventing diabetic nephropathy by blocking epithelial cells phenotype transformation and fibroblast activation (74).

### 4.2 Crosstalk between PTECs and macrophages

Renal fibrosis encompasses different types of inflammatory cells, with macrophages being the primary immune cell involved in renal tissue, ultimately leading to the development of renal fibrosis (100). Macrophages not only detect different inflammatory stimuli but are also affected by diverse cell populations, resulting in a change in the proinflammatory characteristics and the secretion of inflammatory agents (101). Several research studies have indicated that macrophages possess the ability to release pro-fibrotic substances and have a multifaceted impact on the development of renal fibrosis (102). Initially, in response to injury, PTECs release different chemokines and cytokines in the vicinity of peritubular compartments to lure and guide the entry of macrophages into the tubulointerstitial area. Afterward, macrophages become active and generate a blend of soluble substances, which consist of inflammatory cytokines, fibrotic cytokines, and MMPs. In this section, we provide a summary of empirical evidence regarding the paracrine mechanisms that facilitate communication between epithelial cells and macrophages (**Table 2**).

**Table 2:** Summary of mediators that mediate communication between proximal tubular epithelial cells and macrophages during kidney injury.

Responding cells	Target cells	Regulators	Mediators	Experimental models	Disease	Main functions	Upstream/Downstream pathway of regulators	References
2.1 PTECs -Derived Communication with Macrophages								
*2.1.1 Exosomes and Extracellular vesicles*								
PTECs	Macrophages	Upregulate and downregulate miR-374b-5p *in vivo;* overexpression and knockdown SOCS1 *in vitro;* upregulate and downregulate miR-374b-5p *in vitro*	Exosomal miR-374b-5p ↑	IRI model; Hypoxia treated *in vitro*; TKPTS TEC, HEK293T cell, RAW264.7 cell	AKI	Inflammatory response and M1 macrophage activation	**Downstream:** SOCS1 inhibition and inflammatory factor release	(103)
PTECs	Macrophages	Downregulate miR-19b-3p *in vivo*; upregulate and downregulate miR-319b-3p *in vitro*	Exosomal miR-19b-3p ↑	LPS model/ADR model; Mouse tubular epithelial cells; RAW264.7 cell; bone-marrow derived macrophages	AKI CKD	M1 phenotype polarization	**Downstream:** SOCS1 inhibition and NF-κB activation	(104)
PTECs	Macrophages	Upregulate miR-199a-5p *in vivo*; upregulate and downregulate miR-199a-5p *in vitro*; knockdown and overexpression Klotho *in vitro*; knockdown Rab27a *in vitro*	Evs miR-199a-5p ↑	HFD/STZ model; db/db model; HK-2; THP-1	DKD	M1 phenotype polarization	**Downstream:** induce M1 polarization by targeting the Klotho/TLR4 pathway	(105)
PTECs	Macrophages	Overexpression EVs *in vivo*; knock down Rab27a and HIF-1α *in vitro*	Evs miR-155, Evs miR-21, Evs miR-182	db/db model; CD11b^+^ mouse primary cell line; HK-2; THP-1	DKD	Promote macrophage glycolysis; Activate inflammatory response; and promotes fibrosis	**Downstream:** promote the expression of HIF-1α in macrophages, increase glycolysis, and release pro-inflammatory and fibrotic factors	(105)
PTECs	Macrophages	Downregulate CCL2 in mouse tubular epithelial cells;	Exosomal CCL2 ↓	LPS model; 5/6 nephrectomy model; Injection of exosomes through caudal vein; BSA treated *in vitro*; Mouse tubular epithelial cells; HEK-293 cells; RAW264.7 cell	AKI CKD	kidney inflammation	**Downstream:** NGAL in macrophages	(101)
2.1.2 Inflammatory factors								
PTECs	Macrophages	CCR2b inhibitor RS-102895 *in vivo;*	CCL2 ↓	RVH model; Primary renal macrophages;	RVH	Macrophage accumulation and renal atrophy	**Downstream:** Activate the expression of iNOS in macrophages by binding with CCR2 and convert to proinflammatory phenotype	(106)
PTECs	Macrophages	Downregulate IL-1β and IL-1R1 *in vivo;*	IL-1β ↓	db/db model; primary mouse tubular cells; bone-marrow derived macrophages	DKD	Macrophages convert to pro-inflammatory phenotype and cause salt sensitivity	**Downstream:** IL-6 in Macrophage and ENaC activity by binding to IL-1R1 receptor	(107)
PTECs	Macrophages	PTEC -specific knockout of LKB1 *in vivo*; PTEC -specific knockout of PKD1 *in vivo*; Downregulate LKB1 and PKD1 *in vitro*	CCL2 ↑	Ksp-LKB1 mice; Ksp-PKD1 mice; HEK 293T; MDCK cell;	PKD	Activate chemokine signaling to recruit inflammatory cells	**Upstream:** Low expression of ciliary module composed of LKB1 in PTECs **Downstream:** accumulation of CCR2+ mononuclear phagocytes; promoting a ciliopathy phenotype.	(108)
PTECs	Macrophages	MMP-2/9 inhibitor or MMP-9-neutralizing antibody *in vivo*; Downregulate MMP-9 *in vitro*	MMP9 ↓	UUO model; Primary proximal TEC; C1.1 cell; CD11b^+^ mouse primary cell line	CKD	MMP-9-cleaved osteopontin; macrophage infiltration; tubular cell EMT; renal fibrosis	**Downstream:** cleaving osteopontin (OPN)	(109)
PTECs	Macrophages	Upregulate MIF *in vitro*;	MIF ↓	NRK52E;	–	inflammatory cell accumulation and activation	Upstream: IFN-γ	(110)
PTECs	Macrophages	Overexpression and inhibition miR-374a *in vitro* Overexpression MCP-1 *in vitro*	MCP-1 ↑	HK-2 cells; U937 cells	DKD	Promote the activation of macrophages	**Upstream:** miR-374a in PTECs **Downstream:** Induce IL-1, IL-6, IL-8 and TNF- α expression in Macrophage	(111)
PTECs	Macrophages	–	MCP-1 ↑ M-CSF ↑	Unilateral renal vein ligation model;	CKD	Macrophage infiltration; ECM generation	**Downstream:** TNF-α in Macrophage	(112)
2.1.3 Other factors								
PTECs	Macrophages	Overexpression and inhibition sEH in PTECs	sEH	HK-2 cells, RAW264.7 cells,	IgAN	Promotes macrophage infiltration; M1 polarization	**Upstream:** EETs in PTECs **Downstream:** NF-κB pathway induce M1 polarization; PI3K pathway induced M2 polarization	(113)
PTECs	Macrophages	Upregulate miR-219 and downregulate SAP130 *in vivo*; Knock down SAP130 *in vitro;* Upregulate and downregulate miR-219 *in vitro*	SAP130 ↓	C57BL/6J- Mincle^−/−^ mice; UUO model; Cisp-induced AKI; RAW264.7; immortalized mouse tubular epithelial cells	AKI	trigger macrophage activation and necroinflammation	**Downstream:** elicit Mincle activation in macrophages and necroinflammation via the miR-219c-3p-dependent mechanism.	(114)
2.2 Macrophages-Derived Communication with PTECs								
*2.2.1 Exosomes and Extracellular vesicles*								
Macrophages	PTECs	miR-7002-5p inhibitor *in vivo*; GW4869 treatment *in vitro*	Exosomal miR-7002-5p ↓	RAW264.7 cells; Mouse tubular epithelial cells; HG treated with RAW264.7 cells	DKD	Inhibits autophagy; induces inflammation	**Downstream:** ATG9b inhibition and enhanced P62 in PTECs.	(115)
Macrophages	PTECs	miR-195a-5p inhibitor in primary tubule epithelial cells;	Exosomal miR-195a-5p ↓	Cisp-induced AKI model; primary tubule epithelial cells;	AKI	Mitochondrial disorder; induce cell injury in PTECs	**Downstream:** Reduce UQCRC2, mtco1, ATP5b, and Ndufs4 expression in PTECs	(116)
Macrophages	PTECs	Upregulate LRG1 and downregulate TGFβR1 in HK-2 cells	EVs LRG1 ↓	AAN model; HK-2 cells; THP-1 cells; bone marrow-derived macrophages	AAN	Promoted injury, oxidative stress, and apoptosis in PTECs	**Upstream:** IL-1β and CXCL12 in Macrophage **Downstream:** Promote the transcription activity of NOX4 with TGFβR1 dependent	(117)
Macrophages	PTECs	Upregulate Lcn-2 and cisplatin treated with PTECs; IL-10 treated with bone marrow-derived macrophages	EVs Lcn-2 ↑	Lcn-2^−/−^ mice; Primary mouse tubular epithelial cells; bone marrow-derived macrophages	AKI	Macrophage polarization; chelates iron; Promote PTECs proliferation and regeneration	**Upstream:** IL-10 in Macrophage **Downstream:** Induce E-cadherin, N-cadherin and KIM-1 expression in PTECs though chelates iron	(118)
2.2.2 Inflammatory factors								
Macrophages	PTECs	GM6001 treatment in C1.1 tubular epithelial cells	MMP9 ↓	UUO model; C1.1 tubular epithelial cells; primary tubular epithelial cells; J774 macrophages	CKD	Promoted EMT in PTECs;	**Downstream:** Induce E-cadherin、N-cadherin and vimentin expression in PTECs	(119)
Macrophages	PTECs	Silence circACTR2 and up-regulate miR-561 *in vivo*; Overexpress and knock down circACTR2 and miR-561 in Macrophage; Inhibition NLRP3 in Macrophage; Inhibition fascin-1 in PTECs	IL-1β ↑	UUO model; THP-1 cells; HK-2 cells	CKD	Promotes macrophage infiltration; promoted EMT in PTECs; fibroblast activation;	**Upstream:** CircACTR2 promoted the activation of NLRP3 inflammasome and release of IL-1β **Downstream:** Activating fascin-1 to promote fibrosis	(120)
Macrophages	PTECs	Inhibition CX43 *in vitro*;	IL-1β ↓ IL-18 ↓	CX43^-/-^ model; Bone marrow-derived macrophages cells; NRK cells;	AKI	Inflammasome activation; inflammatory cell injury; regulation of intracellular redox status	**Upstream:** Cx43 mediates NLRP3 inflammasome activation **Downstream:** ROS production in PTECs to promote fibrosis;	(121)
2.2.3 Other factors								
Macrophages	PTECs	Downregulate miR-150 *in vivo*; Downregulate miR-150 in PTECs	NA	FA model; HK-2 cells; THP-1 cells	AKI	Macrophage accumulation, tubulointerstitial fibrosis	**Downstream:** MIR-150 activates the JAK/STAT pathway by inhibiting SOCS1	(122)
Macrophages	PTECs	Phorbol 12-myristate 13-acetate, LPS, HAP and IL-4 treated with U937 cells	NA	HK-2 cells; U937 cells;	Urolithiasis	Promotes apoptosis and expression of inflammatory factors in PTECs	**Downstream:** Reduce Bax, BCL2 and CCL2 in PTECs	(123)
2.3 Reciprocal activation loop between PTECs and macrophages								
Macrophages	PTECs	Knockout Prx3 *in vivo*; Downregulation Prx3 *in vitro*	TNF-α ↑ IL-6 ↑ COX2 ↑	C57BL/6J-Prx3^-/-^ model; STZ model; UUO model; Immortalized murine proximal tubular epithelial; RAW264.7 cells	CKD	Promote fibrosis and inflammation	**Upstream:** Prx3 deletion accelerated activation of p-NF-κB leading to the release of inflammatory factors **Downstream:** Promoted EMT in PTECs; fibroblast activation; ECM generation;	(92)
PTECs	Macrophages		MCP-1 ↑ COX2 ↑ ICAM-1 ↑				**Downstream:** ROS production and pro-inflammatory factor secretion in Macrophage	
PTECs	Macrophages	Downregulation TGFβ-R1 in THP-1 cells	Evs LRG1 ↓	HFD+STZ HK-2 cells; primary mouse tubular cells; THP-1 cells; Bone marrow-derived macrophages	CDK	Promote inflammation in Macrophages	**Downstream:** Induce the expression of IL-1β, TNF-αand CXCL12 with TGFβR1 dependent	(124)
Macrophages	PTECs		Evs TRAIL ↓			Injury and apoptosis in PTECs	**Downstream:** Induce injury and apoptosis in PTECs with DR5 dependent	(124)

#### 4.2.1 PTECs-derived communication with macrophages

##### 4.2.1.1 Exosomes and Extracellular Vesicles

MiRNAs are the predominant intracellular communication molecules among the exosomes and EVs derived from PTECs, which induce phenotypic changes in macrophages. Exosomes derived from PTECs have demonstrated the ability to suppress the expression of suppressor of cytokine signaling-1 (SOCS1) in macrophages, resulting in the conversion of macrophages to the M1 phenotype (103, 104). Likewise, miR-199a-5p in EVs originating from human serum albumin-induced PTECs triggered M1 polarization and expedited the advancement of DKD by targeting the Klotho/TLR4 (toll like receptor 4) pathway (92). It has also been shown that PTECs-derived EVs (containing miRNA-155, miRNA-21 and miRNA-182) induce glycolysis in renal macrophages by inducing the overexpressing HIF-1α (105). Macrophages can release factors and glycolytic products that directly contribute to tissue fibrosis when there is an elevated glycolytic flux (105). These studies indicate that PTECs-derived EVs can not only affect the phenotype of macrophages but also affect the metabolic status of macrophages.

In addition to miRNAs, EVs can carry proteins for cell-to-cell information transfer. Chemokine 2 (CCL2), also referred to as monocyte chemoattractant protein or monocyte chemoattractant protein-1 (MCP-1), is an inflammatory factor that has demonstrated a crucial function in the infiltration of macrophages, resulting in tissue damage (125). CCL2 is produced by injured PTECs and transported to macrophages in the form of EVs, which plays a key role in albumin-induced tubulointerstitial inflammation (101). CCL2 is not only transmitted as EVs between PTECs and macrophages but also mediates signal transmission through receptor-ligand binding. Chemokine receptor 2 (CCR2) is the primary receptor for CCL2 and is found in renal macrophages (106). Research has indicated that CCL2/CCR2 signaling enhances the pro-inflammatory characteristics of immune cells in different experimental models of CKD (106, 108).

##### 4.2.1.2 Inflammatory factors

In addition to secreting numerous inflammatory factors, macrophages can also be influenced by inflammatory factors like IL-1β, matrix metalloprotein 9 (MMP9), migration inhibitory factor (MIF), and monocyte chemoattractant protein-1 (MCP-1). IL-1β released from PTECs polarizes macrophages to a pro-inflammatory phenotype, and these cells release IL-6 through an IL-1β/IL-1βR binding mode (107). Notably, this research demonstrated that PTECs rather than macrophages serve as the primary origin of IL-1β. Furthermore, the buildup of IL-6 can result in renal inflammation and salt sensitivity, which exhibits a strong correlation with the development of hypertension. The cellular sources of MMP-9 are associated with different stages of nephropathy. During the initial phase, PTECs serve as the primary source. With the progression of TIF, PTECs, macrophages and myofibroblasts can all produce MMP-9 (109). Additional macrophages may be recruited by MMP-9 derived from macrophages and PTECs macrophages through osteopontin cleavage (126). Inhibition of MMP-9 in the early phase of renal fibrosis and the late phase of nephropathy could decrease tubular cell pEMT (109). Macrophage migration inhibitory factor (MIF) plays a crucial role in both the innate and adaptive immune systems by activating macrophages (127). MIF is enzymatically active, setting it apart from other pro-inflammatory cytokines (128), and has the ability to control cell proliferation and differentiation (129). PTECs have demonstrated the ability of interferon-γ (110) to enhance the production and release of MIF. Monocyte chemoattractant protein (MCP)-1 is a chemokine responsible for attracting macrophages to inflammation areas. Damaged PTECs can also secrete MCP-1, which is negatively regulated by miR-374a (111), leading to increased macrophage infiltration and recruitment in the kidney (112).

##### 4.2.1.3 Other factors

Soluble epoxide hydrolase (sEH) metabolizes epoxyeicosatrienoic acids (EETs) into dihydroxyeicosatrienoic acids (DHETs). It is believed that the function of sEH is the primary factor affecting the availability of EET (130). EET has various biological functions, such as vasodilation and inhibiting inflammation (113). The excessive expression of sEH in PTECs triggers the NF-κB pathway in macrophages, resulting in the activation of M1 macrophages. Conversely, suppression of sEH expression promotes M2 polarization via the PI3K pathway, in the context of IgA nephropathy (113). According to recent research, endogenous damage-associated molecular patterns (DAMPs) have the potential to activate the innate immune system. Endogenous molecules released after cell injury can activate inflammatory pathways during aseptic inflammation (131). Damaged cells release these molecules which then stimulate pattern recognition receptors (PRRs) found on immune cells (132, 133). Mincle, a crucial receptor for recognizing patterns, detects cell death and triggers the release of inflammatory cytokines, leading to the influx of inflammatory cells into injured tissues (134). Sin3A associated protein 130 (SAP130) belongs to the small ribose protein group and exits from injured PTECs. It acts as a recognized natural binder of Mincle in macrophages (135). SAP130 binding to Mincle can drive necroinflammation in AKI (136).

#### 4.2.2 Macrophages-derived communication with PTECs

##### 4.2.2.1 Exosomes and extracellular vesicles

Paracrine communication also occurs during macrophage-derived communication with PTECs. Single-stranded miRNAs are the most extensively studied factors (137). Macrophages can encapsulate miR-7002-5p and miR-195a-5p in exosomes. Specifically, exosomes carry excess miR-7002-5p and inhibit autophagy by targeting Atg9b in PTECs in the presence of high glucose, which subsequently induces tubular dysfunction and inflammation (115). miR-195a-5p is upregulated in a model of cisplatin-induced AKI. Macrophages transport miR-195a-5p through exosomes, resulting in tubular mitochondrial dysfunction and cell damage (116).

Proteins are also important paracrine mediators (138). LRG1 (leucine-rich-alpha-2-glycoprotein 1), also known as leucine-rich alpha-2-glycoprotein 1, has the ability to directly interact with TGF-β receptor 1 (TGF-βR1) and modify TGF-β signaling to produce its effects. Macrophages release extracellular vesicles (EVs) abundant in LRG1, which have been demonstrated to cause injury and apoptosis in PTECs via TGF-β1-dependent mechanisms (117). Following renal injury, macrophages communicate unidirectionally with PTECs, resulting in negative effects. However, a 2020 study revealed that macrophages with an anti-inflammatory phenotype can release macrophage-secreted lipocalin-2 (Lcn-2) EV2.Lcn-2 promotes PTEC regeneration after injury by chelating iron (118). The discovery implies that the type of macrophage affects the inflammatory surroundings and has a crucial part in deciding the course of PTEC restoration (139).

##### 4.2.2.2 Inflammatory factors

Macrophages can release a variety of inflammatory factors, but only a few factors have been reported to act on PTECs. Activated macrophage-conditioned medium is enriched in MMP-9 and can induce EMT phenotypic transformation in PTECs (119). In macrophages, circACTR2 has the ability to attach to miRNAs, stimulate the activation of NLRP3 (nOD-like receptor thermal protein domain associated protein 3) inflammasome, and trigger the secretion of IL-1β. Additionally, in PTECs, the upregulation of fascin-1 expression caused by IL-1β leads to renal fibrosis (120). Disruption of the redox equilibrium in macrophages can partially trigger the activation of the NLRP3 inflammasome, resulting in the release of IL-1β and IL-18 (120). In addition, Cx43 can activate the NLRP3 inflammasome, producing the same effect as CircACTR2 (121).

##### 4.2.2.3 Other factors

In kidney disease, although cellular communication has been extensively studied, many mechanisms remain unknown. MiR-150 was significantly upregulated in PTECs after coculture with macrophages. The presence of this element has the ability to inhibit the SOCS1/JAK (janus kinase)/STAT(signal transducer and activator of transcription) pathway, resulting in a reduction in the infiltration of macrophages (122). PTECs can aggravate apoptosis and proinflammatory factor expression in response to stimulation with conditioned medium derived from macrophages (123). The intermediate conditions in which these phenomena occur require further exploration.

#### 4.2.3 Reciprocal activation between PTECs and macrophages

Communication between PTECs and macrophages is not unidirectional, and feedback can occur. Damaged tubular epithelial cells trigger the activation of macrophages, which subsequently secrete pro-inflammatory cytokines, leading to the induction of apoptosis in the injured cells. Peroxide reductases (Prxs) belong to a group of alcohol peroxidases that function as cellular thiol peroxidases. Among the six members, Prx3 stands out as a distinctive antioxidant (140). It specifically localizes to mitochondria and has a crucial function in preserving mitochondrial reactive oxygen species (ROS) (141), while oxidative stress caused by ROS serves as a significant mediator of CKD. The acceleration of CKD through positive feedback between macrophages and PTECs has been demonstrated by the removal of Prx3 (92). The deletion of Prx3 in PTECs accelerates the development of fibrosis and inflammation, which are accompanied by increased oxidative stress in the mitochondria. Furthermore, the deletion of Prx3 triggers the activation of macrophages, resulting in the increase of pro-inflammatory cytokines and the expedited release of pro-inflammatory and profibrotic cytokines by PTECs. Similarly, there is a negative feedback loop between PTECs and macrophages. Several research studies have indicated that EVs derived from PTECs stimulate the inflammatory macrophage phenotype and trigger the secretion of EVs derived from macrophages (101). Moreover, EVs trigger programmed cell death in PTECs. Notably, EVs enriched with leucine-rich alpha-2-glycoprotein 1 (LRG1) stimulate macrophages through TGF-βR1-dependent mechanisms, while EVs enriched with tumor necrosis factor (TNF)-related apoptosis-inducing ligand (TRAIL) trigger apoptosis in injured PTECs through death receptor 5 (DR5)-dependent mechanisms (124). To sum up, the reciprocal communication between PTECs and macrophages plays a crucial role in the development and upkeep of homeostasis in mammals, and it has the potential to expedite the advancement of kidney disease.

### 4.3 Crosstalk between PTECs and endothelial cells

Most nutrient exchange processes occur in the adjacent tubular epithelium, which is tightened by the peritubular capillary network in the renal cortex. Substances and water are absorbed again from the PT, absorbed by the vascular system, and returned to the circulation, while the substance is also secreted into the PT. Nephrogenesis and the assembly of microvessels are closely linked in the developing kidney (142). In chronic kidney disease, the separation of endothelial cells, which are thin cells positioned amidst the vessel wall and the flow of blood, results in a scarcity of blood vessels and inadequate blood provision, thereby exacerbating renal fibrosis. The complex communication networks between epithelial cells and endothelial cells have been demonstrated to impact angiogenesis, regulation of solutes, and the high expression of certain genes (143).

The gene family of vascular endothelial growth factor (VEGF) is composed of VEGF-A, VEGF-B, VEGF-C, VEGF-D, VEGF-E, and placental growth factor (PlGF). Originally identified as a factor that increases the permeability of blood vessels (144), VEGF-A plays a crucial part in the development of blood vessels by stimulating angiogenesis. VEGF-A is found on various types of cells, such as PTECs, and its receptor (Kdr/Vegfr2) is believed to be primarily situated on peritubular capillary endothelial cells in the vasculature behind the glomerulus (145). The demonstration of VEGF’s involvement in preserving elevated permeability and widespread angiogenesis in various tumors and endocrine glands has been established (146). EVs secretion by upregulating COX-2 expression endothelial cells have been shown to increase HIF-1α expression and VEGF-A production in PTECs (147). PTECs induce VEGF-dependent capillary vessel formation in cocultured endothelial cells, and hypoxia further increases angiogenesis. Further studies showed that knockdown of the VEGF-A gene in PTECs resulted in selective shedding of peritubular microvessels. The specific mechanism of VEGF-A in renal PTECs is achieved by mediating crosstalk in VEGFR2-expressing endothelial cells. This form of communication is also linked to a significant rise in Epo production within the kidney, resulting in significant erythrocytosis (145).

TNF-α receptors (148) have been observed in the S2 and S3 sections of proximal tubules, indicating the presence of PTECs. When cocultured with endothelial cells (HGECs), epithelial cells may produce inflammatory mediators by sensing TNF-α released from HGECs (149). Furthermore, transcriptomic analysis showed that differentially expressed genes in PTECs were mainly enriched in extracellular matrix (e.g., lysyl oxidase), intercellular communication (e.g., IL-6), and transport pathways (e.g., neutrophil gelatinase-associated lipocalin 2) in coculture systems involving endothelial cells. On the other hand, the genes that were expressed differently in endothelial cells mainly acted as regulators of epithelial cell function, such as proteins in the extracellular matrix (e.g., collagen I, III, IV and V, laminin IV) and cytokines/growth factors (e.g., HGF, VEGF-C, etc.) (150). The discovery reveals an intricate communication system between microvascular endothelial cells and epithelial cells that ultimately affects PTECs function.

## 5 Conclusion

**Figure 3:**
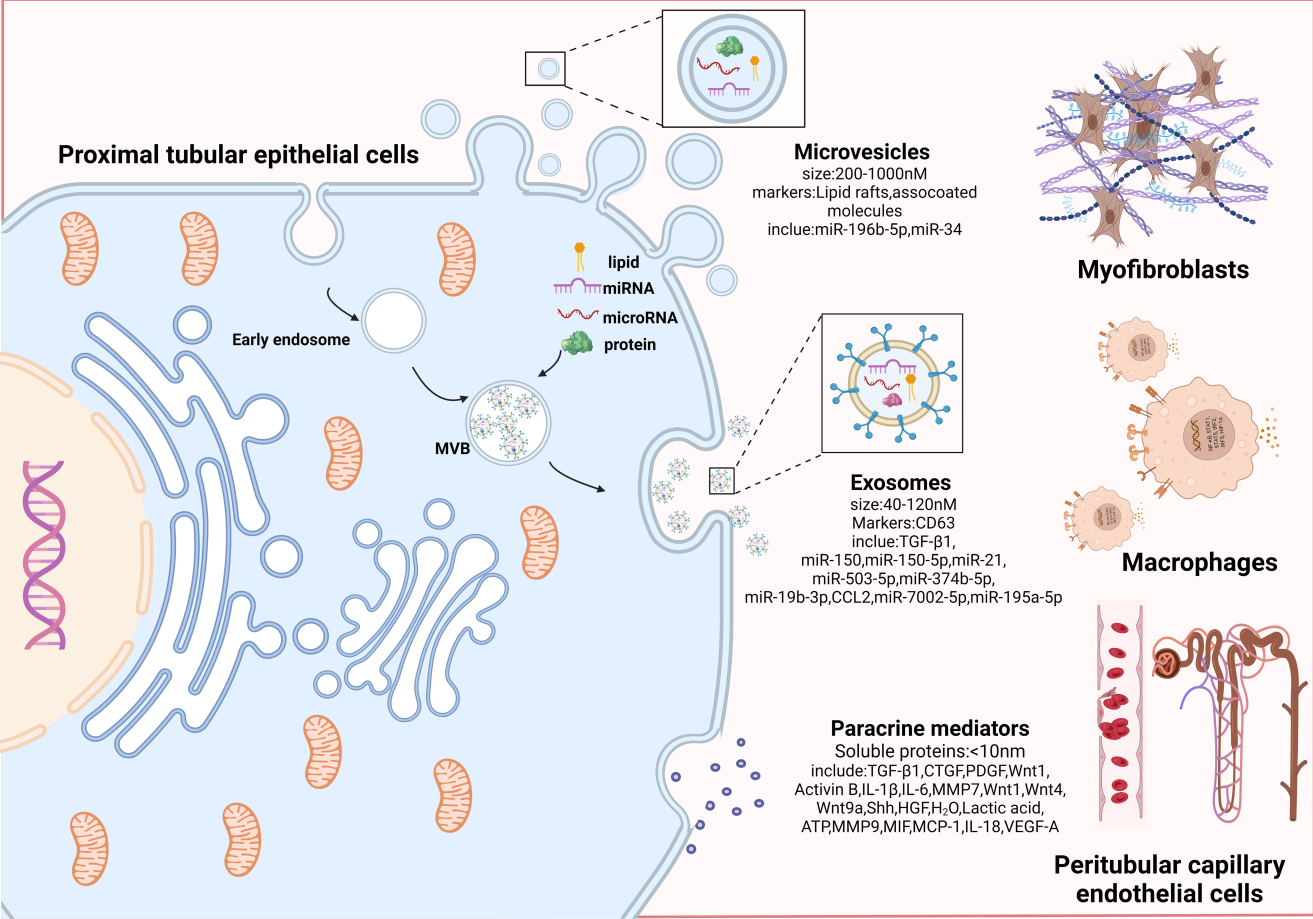
Proximal tubular epithelial cells crosstalk with other interstitial cells to promote progressive tubulointerstitial fibrosis. An overview of paracrine manner. Paracrine signals can be divided into three categories according to effector substances and secretion particle size: microvesicles, exosomes, and paracrine mediators. Microvesicles (200-1000nM in size; markers: Lipid rafts and associated molecules) are shed directly from the plasma membrane by outward budding; exosomes are small membrane particles (40 to 120nM in size; markers: CD63) released by fusion of multivesicular bodies with the plasma membrane, which encapsulate miRNAs and proteins internally; paracrine mediators are the secretion of various paracrine factors composed of soluble proteins by fusion of paracrine granules with the plasma membrane. Among them, microvesicles and exosomes are collectively referred to as extracellular vesicles (EVs), because they can efficiently carry bioactive molecules including mRNA, proteins, and microRNAs to facilitate transfer of genetic information between different cells. PTECs affect myofibroblasts activation and ECM production through bioactive substances (miR-196b-5p, TGF-β1, etc.). PTECs mediates macrophage activation and accumulation by secreting inflammatory mediators (CCL2, MMP9, IL-18, etc.). Communication between PTECs and epithelial occurs via paracrine secretion of VEGF-A. Combined with the above intercellular communication medias centered on PTECs, they jointly lead to the progression of TIF.

CKD is most commonly attributed to diabetes, hypertension and AKI.As the early CKD caused by these diseases are often asymptomatic, the Kidney Disease Improving Global Outcomes (KDIGO) conference emphasized the importance of early identification and early treatments of CKD in order to delay the advancement of the disease (151). Hence, it is imperative to ascertain the initial cellular occurrences that trigger renal fibrosis and discover novel targets for therapeutic intervention. The high energy consumption and metabolic characteristics of PTECs established their pivotal role as an initial cell affected in the early phase of renal injury. To survive, PTECs undergo shifts toward glycolysis enhancement, pEMT and cellular senescence. These transitions can be either positive or negative depending on their timing and duration. Except for HGF and MIF, the majority of active compounds facilitate this harmful communication between the damaged epithelial cells and interstitial cells in CKD (**Figure 3**). Therefore, understanding the timing of targeted PTECs phenotypic shifts and clarifying the mechanism of the intricate system of communication may be the key to preventing and treating nephropathy in the future.

## Author contributions

CG: Conceptualization, Data curation, Formal analysis, Funding acquisition, Writing – original draft. YC: Data curation, Formal analysis, Visualization, Writing – original draft. MJ: Software, Writing – review & editing. JY: Writing – review & editing, Investigation. JZ: Data curation, Writing – original draft. YT: Data curation, Visualization, Writing – review & editing. JD: Supervision, Writing – review & editing. LL: Funding acquisition, Project administration, Writing – review & editing.
